# Co-designing a framework to communicate patient-centred outcomes in palliative care: involving patients and the public to reframe understanding

**DOI:** 10.1186/s41687-026-01019-y

**Published:** 2026-02-21

**Authors:** Mevhibe B. Hocaoglu, Adejoke Oluyase, Deb Smith, Rashmi Kumar, Sarah Perman, Matthew Maddocks, Sian Best, Chloe Nast, Sabrina Bajwah, Katherine E. Sleeman, Irene J. Higginson

**Affiliations:** 1 Cicely Saunders Institute of Palliative Care, Policy & Rehabilitation, King’s College London, London, UK; 2 Cicely Saunders Institute Public Involvement Forum, London, UK; 3 Hertfordshire County Council, Hertfordshire, UK; 4 King’s College Hospital NHS Foundation Trust, London, UK

**Keywords:** Palliative care, Communication, Stakeholder participation, Patient outcome assessment, COVID-19, Co-design, Knowledge translation, Public engagement, Patient-centred care

## Abstract

**Background:**

Palliative care provides person-centred support to alleviate suffering in people with serious illnesses. However, common misconceptions that it is only for end-of-life care often delays timely access. This study aimed to co-design a framework and use it to co-produce resources that enhance public communication and engagement around patient-centred outcomes in palliative care—a field frequently overlooked despite its broader role in improving quality of life throughout the course of illness.

**Methods:**

Two online co-design workshops, guided by the Knowledge-to-Action framework, engaged patients, public and senior leaders from health and social care systems. Public participants were recruited from the Cicely Saunders Institute Public Involvement Forum, while senior leaders were recruited from integrated care systems and their partners. Both workshops focused on presenting evidence from the CovPall study—a national multicentre cohort study that examined symptom changes in 572 severely ill or dying patients with COVID-19 who received palliative care. As many patients were too unwell to self-report due to the severity of illness, a proxy-reported version of the Integrated Palliative Outcome Scale adapted for COVID-19 was used. Workshops shared these findings and explored effective communication strategies, dissemination formats, stakeholder engagement, and ways to evaluate the impact of such communication. Discussions were analysed using thematic analysis. Results: Eighteen members of the public—including patients, carers, and individuals with lived experience of both roles—along with two local board members and one representative from a statutory health and social care body, participated and were actively involved in developing the framework and co-producing outputs through structured small-group discussions. Key findings highlighted the value of using clear, accessible, and culturally sensitive formats—such as short films and infographics—to reframe public understanding of palliative care as a holistic approach that addresses complex needs beyond just end-of-life care. Participants emphasised the importance of using insights from patient-centred outcomes and Patient-Reported outcome (PRO) data to build workforce capacity, improve services, and strengthen the connection between clinical and personal outcomes to maximise impact. As part of the study, we co-produced two resources that applied this framework in practice, demonstrating its potential to support more effective communication and public engagement. While based on COVID-19 outcome data, the co-produced framework and resources were developed to support communication that is relevant across broader palliative care settings.

**Conclusions:**

This study found that communication of patient-centred outcomes and Patient-Reported outcome (PRO) evidence can be strengthened through meaningful patient and public involvement and engagement (PPIE). This approach helps to reframe public understanding of palliative care, highlighting its broader relevance beyond end-of-life settings. The findings show how PPIE can amplify the impact of patient-centred research by engaging wider audiences, promoting timely access to care, and supporting better use of data. While developed in the context of palliative care, the framework offers transferable strategies for communicating complex outcomes in other often misunderstood or stigmatised areas, such as mental health and dementia care.

## Background

Palliative care is crucial for people with complex needs, symptoms and living with serious illnesses [1]. However, the varied terminology used to describe palliative care throughout the illness trajectory—such as supportive care, hospice, and end-of-life care—can create confusion among patients and public, often deterring timely access [2]. Mobilising evidence is essential to influence practice and integrate palliative care into the management of complex needs, symptoms and illnesses [3] and improve public understanding of palliative care [4]. Frameworks and resources are vital for clear public communication, especially where misconceptions delay care [5, 6].

The COVID-19 pandemic highlighted the vital role of palliative care in managing complex and distressing symptoms in life-threatening COVID-19. To better understand this role, we conducted the CovPall study — a national multicentre cohort study that evaluated palliative care provision across the UK during the pandemic [7]. The study identified key challenges, including gaps in service integration and access to care, and highlighted innovations such as frugal approaches to symptom management and streamlined care pathways. These findings informed the basis for this co-design study. Patient-centred evidence from CovPall, published elsewhere, used the proxy-reported Integrated Palliative care Outcome Scale – COVID version (IPOS-COV) to assess symptom trajectories in 572 patients who were severely ill or dying from COVID-19 [8]. Because many patients were too unwell to self-report, the use of a proxy version was essential to ensure their experiences were captured. The study found that many symptoms—particularly breathlessness, agitation, anxiety, and pain—improved during palliative care, although drowsiness worsened [9]. It also highlighted the importance of timely referral to palliative care, with severe breathlessness and multiple comorbidities serving as potential indicators of deterioration [8, 10]. The CovPall Study, integrated patient and public involvement, engagement, and consultation throughout all stages, from inception to dissemination [11].

Building communities and supporting open collaboration is key to effective patient and public involvement and engagement (PPIE) in research [12]. PPIE has been instrumental in the development of patient reported outcome measures (PROMs), which capture what matters most to patients [13]. New frameworks to incorporate public involvement in patient reported outcome measure (PROM) development [14] and wider patient-centred outcomes research has emerged [15]. Patient-centred outcome measures (PCOMs) refers to tools that capture outcomes that matter most to patients, and includes PROMs as a key subset [15]. PPIE is vital for adding value to research, enhancing reporting, avoiding ‘research waste’ [16], improving study design, boosting enrolment, and increasing patient participation [17]. PPIE bring value to patient-centred outcome research, addressing challenges in implementing PCOMs in experimental studies [18], and in developing core outcome sets. Recognising the pivotal role of PPIE in patient-centred research, our study aimed to co-design a framework and co-produce resources with patients, the public, and senior leaders to enhance communication and engagement with patient-centred outcomes evidence, focusing on the Integrated Palliative/Patient Outcome Scale (IPOS) proxy version adapted for COVID-19.

## Methods

### Study design

This qualitative study used a co-design approach to develop a framework and co-produce resources to improve public communication of patient-centred outcomes in palliative care. The study was guided by the Knowledge-to-Action (KTA) framework [19], with evaluation planning informed by the RE-AIM framework [20]. The consolidated criteria for reporting qualitative research (COREQ) were followed to ensure transparency and comprehensibility [21]. Based on this framework, the study co-produced two resources.

### Setting and participants

Two online co-design workshops were conducted. The first workshop engaged members of the public, while the second involved senior leaders within health and social care systems, including Integrated Care Systems (ICSs) [22] and their system partners.

Public Participants: An email invitation was sent to all ~ 240 members of the Cicely Saunders Institute Patient and Public Involvement Forum. The forum— an online, moderated community of patients, carers, and members of the public with diverse lived experience of palliative care, rehabilitation, and related fields is hosted by the Cicely Saunders Institute at King’s College London [23].

The forum is overseen by a PPIE lead and supported by two PPIE coordinators, who manage membership, verify lived experience as self-reported by members at registration, and facilitate meaningful and inclusive involvement. Forum membership is open to anyone with an interest in palliative care, including patients, carers, researchers, clinicians, and other professionals. While ensuring full representativeness is challenging, efforts are ongoing to broaden participation. New members are asked to provide optional demographic information and to state their reason for joining, which helps inform moderation. All membership requests undergo a two-stage screening process: an initial filter to remove spam, followed by review and approval by the moderators. Collected demographic data are used to identify gaps and improve the diversity and representativeness of the forum community. The forum provides a platform for members to contribute to research through involvement in study design, conduct, and dissemination.

We sought 15–20 participants with lived experience of advanced illness or of caring for someone with a life-limiting illness. The invitation strongly encouraged members from ethnic minority backgrounds and those with lived experience of social and economic deprivation to consider participating. Lived experience was self-reported through forum registration and reconfirmed at consent. In total, 23 forum members expressed interest, and 18 attended the workshop.

Senior Leads: We identified and emailed senior representatives of Integrated Care Boards (ICBs)—regional bodies within England’s National Health Service (NHS) responsible for planning and coordinating health and social care services—and their system partners. These system partners included local government authorities and organisations from the voluntary, community, faith, and social enterprise sectors—that is, non-governmental organisations, charities, religious organisations, and mission-driven businesses providing social support or care services. Contact details were sourced from publicly available websites. We invited four members from two ICBs and their partners in London and the South East and East of England. We also sought recommendations from the core project group and local organisations. Due to prior commitments, we were advised that attending planned routine meetings might be more feasible for these individuals. Ultimately, two local board members and one representative from a statutory health and social care body participated.

### Data collection

Workshops: Two co-design workshops were held online in English using video conferencing software. Each session lasted around 2.5 h and included:

(i) A presentation of key evidence from the CovPall study, focusing on evidence around management of complex symptoms based on IPOS-COV reported elsewhere [8, 9].
(ii) Small group discussions in breakout rooms, facilitated by the researchers, using the following prompts:
  How can we rephrase and express the evidence to effectively reach the public and senior leads within the new health and social care structures (especially ICBs and their partners), ensuring that underserved communities can benefit from palliative care?
  Which formats/media would be most accessible and have the furthest reach?
  What opportunities (key people, organisations) should we consider in making this evidence accessible? What strategies can ensure its reach? How do we ensure our resources reach communities with the greatest needs and the senior leads, local authorities, and Voluntary, Community, Faith, and Social Enterprise (VCFSE) - a diverse range of non-profit organizations that play a crucial role in providing services and support within communities - organisations serving these communities?
  How will we know if the evidence has been received and has made an impact? What actions do we want to see in the public, ICBs, and local authorities we aim to reach?
(iii) A plenary session for feedback and synthesis of key points.

### Data analysis

Approach: Transcripts were analysed using thematic analysis [24] to identify strategies and suggestions for improving communication and integration of palliative care evidence.

Process: Discussions were audio-recorded and transcribed verbatim for analysis by two researchers (MBH and AO). One researcher (MBH) conducted an initial coding of all transcripts, identifying broad themes inductively. The preliminary coding schema was discussed with and refined by the core project team, including PPIE members. Patterns and relationships in the data were explored to identify strategies for maximizing the impact and reach of patient-centric evidence. Participant quotations were selected to illustrate the identified themes.

### Researcher characteristics & reflexivity

The co-design workshops were facilitated by two female postdoctoral research associates in palliative care (AO and MBH), both of whom are first-generation migrants born outside the UK, with lived experience as women from racially minoritised communities. Both researchers had previous experience working with some, but not all, of the patient and public involvement members. The researchers played key roles in the original CovPall study, with AO leading service-level and MBH leading patient-level data collection and analysis [7].

### Patient and public involvement

Recognising the importance of incorporating diverse perspectives, we adopted a layered approach to patient and public involvement (PPIE) in this study. Two experienced PPIE contributors, DS and RK—both of whom have dual lived experience as informal carers and as patients—served as core members of the research team. They were involved throughout the project, from study design to data interpretation and dissemination. In addition, we conducted two co-design workshops that included a broader group of patients and carers with varying levels of experience in PPIE, including individuals entirely new to research involvement. These wider contributors provided valuable input during the co-design phase, particularly in refining communication strategies and ensuring practical relevance. This inclusive approach allowed us to benefit from sustained input by experienced PPIE members while also creating space for new voices, supporting capacity building and inclusivity within the PPIE community. We believe this model is particularly well-suited to short-term projects and recommend its wider adoption. For longer-term initiatives, we suggest integrating a mentorship component, enabling experienced PPIE members to support those newer to involvement.

### Ethical considerations

Ethical approval for the co-design workshops was obtained from the King’s College London research ethics committee (LRS/DP-22/23-36449). All participants provided informed consent, and confidentiality was maintained throughout the study.

## Results

### Participant characteristics

Eighteen patient and members of the public participated in the first co-design workshop. Table 1 presents an overview of their self-declared background, experiences, and motivations. The transcripts from this group are labelled as ‘PPIE-F’ or ‘PPIE-M’, for F for female, and M for male participants.

**Table 1:** Overview of members of public who have participated in the co-design workshop (*n* = 18)

Participant Number	Gender	Lived Experience			Ethnic Group	Lived Experience of Deprivation	Region
		*Carer*	*Patient*	*Advocate*			
1	Male	Yes			British Asian	None Declared	
2	Female				British Black Caribbean	None Declared	Cornwall
3	Male		Yes		White British	None Declared	Bristol
4	Female	Yes			White British	None Declared	
5	Male	Yes			White Other (Polish)	Family with lived experience of real financial hardship and deprivation	
6	Male	Yes			British Asian	None Declared	London
7	Female	Yes			British Asian	None Declared	Hampshire
8	Male		Yes		White British	None Declared	
9	Male	Yes			Asian British Pakistani	None Declared	
10	Male		Yes		White British	None Declared	London
11	Female			Yes		None Declared	London
12	Female	Yes	Yes	Yes	Asian British Chinese	None Declared	Manchester
13	Male		Yes		White Scottish	None Declared	Scotland
14	Female		Yes		White British	Socio-economically disadvantaged	
15	Female		Yes		White British	None Declared	
16	Female	Yes			Asian British Indian	None Declared	London
17	Male	Yes			Black (Muslim)	None Declared	
18	Female		Yes			None Declared	Lancashire

The second co-design workshop was run with two members representing the South East and East of England integrated care systems (Integrated Care Boards and Integrated Care Partnerships, UK), and one member of an independent statutory body overseeing health and social care. The transcript excerpts from this group are labelled as ‘ICB&P’. Participants all attended the workshops after providing informed consent. The workshops lasted 2.5 h each.

Participants highlighted the critical need for improved public understanding and awareness of palliative care, the importance of effective communication and engagement strategies tailored to diverse audiences, and the value of patient-centred outcomes in demonstrating the benefits of palliative care.

Within the identified themes, we compared similarities and differences in perspectives between patient and members of the public and local care board representatives.

### Themes

#### Reframing palliative care through patient-centric evidence

Participants consistently highlighted the need to challenge the prevailing misconception that palliative care equates solely to end-of-life care. They recognized the potential of evidence to reframe this narrative, showcasing palliative care’s broader scope. This includes managing complex symptoms, addressing comorbidities, and enhancing quality of life for individuals with serious illnesses, regardless of prognosis. A PPIE member emphasized the importance of demonstrating how palliative care “embraces complex symptoms in comorbidities” (PPIE-F1), extending beyond “imminent death and dying.” Others echoed this sentiment, stressing the need to dispel the misconception that “palliative means death straight away” (PPIE-M2) and acknowledge the widespread lack of understanding about its true purpose and benefits, explaining that “…a lot of people just don’t know what that [palliative care] is, what that means, it is assumed that people know it and they probably don’t” (ICB&P 2). Another ICB&P participant said ‘we need perhaps need to go back to basics and say what is palliative care.’ (ICB&P 1).

#### Accessible language

The importance of clear and accessible communication emerged as a central theme. Participants stressed the need to avoid clinical jargon and use language that resonates with the public, making information about palliative care easily understandable and relatable. They emphasized that effective communication is key to dispelling misconceptions and promoting wider acceptance of palliative care services. As one participant described, “the messages need to be framed in a nonclinical language, what is palliative care and why is it important” (ICB&P 1). For example, a participant explained, ‘…we have the term dying matters… turn the words around and call [it] matters around dying … all these matters that you need to consider around dying’ (PPIE-M6).

#### Promoting timely access and evidence-based advocacy

Participants advocated for promoting earlier access to palliative care, leveraging patient-centric evidence to highlight its benefits. They emphasized the positive impact of palliative care on quality of life and symptom management, as exemplified by the quote, “The quality of life can be improved if they [public] access palliative care” (PPIE-F8). Furthermore, participants stressed the urgency of facilitating timely referrals and expanding the reach of palliative care services to a larger proportion of patients, with one participant stating, “Evidence of the positive effects of being referred and treated by palliative care and we need to get people into palliative care much, much sooner and for a higher proportion of patients” (ICB&P 2). Participants highlighted the need to utilize patient-centric evidence to not only showcase the benefits of palliative care but also advocate for its timely and equitable access.

#### Addressing diverse needs and cultural sensitivity

Recognizing the diverse needs and cultural backgrounds within the community, participants emphasized the importance of tailoring communication and engagement strategies. They highlighted the need to be culturally sensitive and address specific concerns related to symptom management and comorbidities in different ethnic groups and subgroups, ensuring that information about patient-centric evidence is accessible and relevant to all:

Challenge is how to adapt that same message to be acceptable to the ethnic groups and subgroups the message about breathlessness, agitation, and comorbidities (ICB&P 2)

Participants stressed the importance of prioritising outreach to underserved communities, recognizing that certain groups may have “a lesser understanding” of palliative care and are “much less likely to go into Hospice or go into care homes at all,” resulting in them “completely missing out on not only what it is, but also how it could benefit them” (ICB&P 3). Another participant agreed that patient-centred outcomes evidence can play an important role in understanding “You know where there’s inequalities and … there are groups that don’t access palliative care as readily as others” (ICB&P2). Language may not be the only barrier as a public member also highlighted that ‘… it’s not just people from the [non-English speaking] minority communities’ (PPIE-M1) that may struggle to understand what palliative care is and access it, but we need to consider other areas and communities with poor access.

#### Translating evidence into actionable insights

Participants expressed a strong desire for clear, actionable learnings that go beyond the mere presentation of data. They emphasized the importance of translating patient-centric evidence into practical strategies and interventions that can be readily implemented to improve access to and delivery of palliative care services. As one ICB&P representative said, “…include ideas of actions with points to take forward what to do with this evidence, besides presenting the data, key takeaway points” (ICB&P 3), however realistic goals and recognition of opportunities are important ‘that we are not there to educate the entire nation in one go … educate them when the need arises’ (PPIE-F2). This highlights the need to bridge the gap between research findings and real-world implementation, ensuring that patient-centric evidence informs concrete actions to enhance palliative care provision.

#### Patient-centred outcomes to improve care – learnings from the pandemic

The COVID-19 pandemic served as a stark reminder of the critical role of palliative care, particularly in managing the complex needs of severely ill patients. As one participant poignantly reflected, “a lot of people had very bad deaths” (PPIE-M6), highlighting the urgent need for improvement in this area. This experience underscored the value of patient-centred outcomes as a powerful tool for understanding and addressing the suffering associated with serious illness.

Participants emphasized the importance of learning from this crisis, with one ICB&P representative stating, “not losing sight of the importance of palliative care in a pandemic is an important message” (ICB&P 1). Another ICB&P member highlighted the potential impact of patient-centred outcomes on future care, stating the evidence showing changes in patient outcomes during care as, “…these are important findings and if it…makes a difference to that degree to patients who are in extreme distress at the end of life and their families, we really should be making sure that palliative care is recognised as a key part of our…treatment and care services…” (ICB&P 2). Participants collectively expressed a desire to “move forward” and “make things better for people” (PPIE-M6) by incorporating patient perspectives and utilizing patient-centred outcomes to inform both future pandemic planning and routine care practices. This approach ensures that the needs and experiences of patients remain central to delivering compassionate and effective palliative care, even beyond the context of a global health crisis.

#### Need for integration of PRO data with clinical data

Differently to the PPIE members, senior leads in social and health care expressed interest in understanding whether patient-centred outcomes, for example, how breathlessness affects the person correlates with clinical measures such as oxygen levels (ICB&P 2). Demonstrating such alignment not only enhances the credibility of PROs but also enables clinicians to utilize patient perspectives in conjunction with clinical data, leading to more informed and personalized care. By bridging the gap between the subjective experience of symptoms and their objective measurement, patient-centred outcomes can become a powerful tool for improving both clinical decision-making and patient empowerment.

#### Broader applicability of PCOM evidence across care settings and conditions

ICB&P members expressed a keen interest in exploring the broader applicability of the study’s findings beyond the acute hospital setting, and while this topic was very briefly brought up by PPIE members, for example a participant said they would want to understand how ‘…we extrapolate this [understanding/data] into current day-to-day management of palliative patients’ (ICB&P 2). One participant highlighted the potential value of translating these insights to community settings, stating, “… it will be interesting to see if this is in any way useful to say to people in the community, particularly places like care homes and so on” (ICB&P 2). The participant also questioned whether such evidence could be used in care homes to identify “patients at risk of dying imminently” or those requiring “more urgent attention” (ICB&P2). Furthermore, the potential for patient-centred outcomes to inform resource allocation and reduce unnecessary hospital admissions was also recognized.

The participants agreed the importance of ensuring that palliative care evidence and best practices, particularly those derived from patient-centred outcomes, are disseminated and implemented across the entire care continuum, including community settings like care homes. ICB&P members acknowledged the challenge of ensuring research findings are relevant and adaptable across diverse settings. However, they expressed optimism that “if the message is right, it will” (ICB&P 2) resonate and be applicable in various contexts, suggesting that well-framed patient outcomes evidence can transcend specific settings and populations. This highlights the importance of focusing on core principles and actionable insights that can be tailored to meet the unique needs of different communities and healthcare systems.

#### Patient-centred outcomes evidence to build capacity

While the potential for cost savings associated with reduced hospital admissions was acknowledged, participants expressed concerns about framing the value of PCOM evidence solely in economic terms, fearing it could be perceived as simplistic. Instead, they emphasized the need to prioritize patient-centred care and leverage PCOM evidence to empower frontline staff through education and practical tools. This could involve developing easily accessible educational resources for care home staff and focusing on “professional development” (ICB&P 1) opportunities. Participants also expressed a desire for user-friendly tools to aid in interpreting PRO data, such as “a formula that I can give very simply to frontline staff” (ICB&P 2) to guide decision-making regarding palliative care referrals. These suggestions highlight the potential of PCOM evidence to not only enhance the quality of care but also equip healthcare professionals with the knowledge and tools to make informed, patient-centred decisions that transcend purely financial considerations.

### Formats and dissemination

Participants suggested using accessible formats such as infographics and short films for the public, and concise slide decks for health and social care leads, with links to further information. They also recommended disseminating these resources in various public spaces (e.g., GP waiting rooms, libraries) and incorporating them into online training programmes. Key stakeholders for dissemination were identified, including adult social care departments, Integrated Care Partnerships, Healthwatch England, networks of care workers and care homes, bereavement associations, and community groups. Integrated Care Partnerships play a vital role in improving access to care. They include clinical managerial politicians, foster collaboration among diverse stakeholders, bringing together individuals with expertise in health and social care leadership, with a history of innovative work and collaboration, particularly around end-of-life and advance care planning, demonstrating the potential of these partnerships to drive meaningful change in healthcare delivery.

### Framework and resources

Table 2 shows the synthesis of learnings to inform a framework for improving communication and engagement with patient-centred data, linking co-design insights to practical actions in palliative care and outcomes research. The framework also describes how it informed the design of an infographic on palliative care titled ‘Palliative Care for the Treatment of Complex Symptoms’ [25], and a short film on palliative care and its role in management of complex symptoms [26] following cycle of review by the core project team.

**Table 2:** Framework for enhancing communication and engagement with patient-centred data, showing how co-design insights informed actionable steps for palliative care and broader patient-centred outcome research

Theme	Co-Design Insight and Actionable Steps		Quotes from Workshops	Notes on the Infographic & Film
	Other fields	Palliative Care		
Framing & Reframing the Context and Concepts	Co-design clear, plain-language definitions of key concepts relevant to the area or context, ensuring they are meaningful and accessible to diverse audiences.	Frame palliative care as specialist support for managing serious illness and complex symptoms, not just end-of-life care or COVID-specific care.	‘…we raise awareness of what palliative care is… and then we start asking [the] question … about the underserved part of the population’ (PPIE-M1) ‘… not every community or every group know what palliative means or don’t use the word palliative’ (PPIE-M2) ‘…palliative, end of life being lumped together is not good’ (PPIE-M5) ‘… we need perhaps need to go back to basics and say what is palliative care… that we’re not after cure or we’re after symptom control and things like that…’ (ICB&P1)	Infographic introduces palliative care as ‘*treatment of complex symptoms*’, ‘*expert symptom management*’ ‘complex science which will be able to look after you better’ Film scenes show palliative care at home, with carers, friends nurses, and other team members, the patient sitting upright in her own bed, challenging the view that palliative care happens at hospital, and people receiving it are severely ill.
Language & Format	Explore both traditional (e.g. posters, leaflets in waiting rooms, libraries, post offices) and modern formats (e.g. videos, animations, infographics) to communicate key concepts. Co-design materials so that all formats use plain, conversational language, an approachable and non-patronising tone, and are accessible and engaging for diverse audiences. Ensure the resources are easily discoverable.	Co-design short, accessible materials (e.g. videos, infographics) that start with a clear, plain-language explanation of palliative care as support for managing complex symptoms. Use conversational tone, functional subtitles, and inclusive voiceovers. Limit main content to essential information to avoid overload, but provide clear, optional pathways for people to access more detailed information if they choose — recognising and supporting individual agency.	‘… I think you can’t be a better place than a library…You’ve got YouTube, you’ve got Tick Tock…I’m someone that’s got dyslexia and I’m better watching something than trying to read it.’ (PPIE-F4)’ …a video, I think it is very useful … and then how you drive people to it … for them to see it, that’s another part of the equation’ (PPIE-F5) ‘. ‘...video is a great, powerful way of reaching out and getting the message out and having multiple videos. With functional subtitles with audio. With visual it means we can reach out to those with hearing impairments…sometimes if people can see things and watch things, they can pick up things, and if their English is not the best… we need to think about being very creative to promote inclusivity.’ (PPIE-M4) ‘…when you see the word palliative, it should never be without a subheading to ease complex symptoms’ (PPIE-F1) ‘They want three slides maximum… and if you want to look at the third slide with the graph and the and the table on it.’ ICB&P1 ‘… put posters up the GP’s, … we put it up in the emergency halls, we put it in the hospitals … it’s an old old-fashioned, but people are sitting in waiting rooms with nothing to do.’ PPIE-M3	Infographic uses “breathlessness” not “dyspnoea”; simple phrases replace technical terms. Film narration uses a Northern accent, chosen by PPIE as friendly and non-patronising. Infographic and animation as accessible formats.
Access to Timely and Appropriate Care	Patient-Centred Outcome Measures (PCOMs) help structure and clarify the patient’s overall situation by systematically capturing their symptoms, concerns, and priorities. They support professionals to understand what matters most to the patient and guide appropriate care and communication.	In palliative care, where decisions about referral and support can be complex and difficult to communicate, Patient-Centred Outcome Measures (PCOMs) help structure and clarify the patient’s situation by systematically capturing their symptoms, concerns, and priorities at first point of contact. They support professionals in understanding what matters most to the patient and in guiding timely, appropriate care and sensitive communication.	’Your first point of contact is your General practitioner …if a person comes in and produces a certain scenario … say looking at all your symptoms, we are going to refer you to palliative care and then say that palliative care is not only for the dying or … end of life…[it] actually deals with your type of case…’ PPIE-F1	In the infographic, it is stated that palliative care ‘can start early if the symptoms are complex’. …the film emphasizes that palliative care is to address complex physical and emotional symptoms.
Diverse Needs	Outputs should reflect diverse communities and team members and aware of diverse needs	Use inclusive content, format and show multidisciplinary care, recognizing diversity of needs and ways	‘…what we come across a lot is that there are particular communities … are much less likely to go into Hospice or go into care homes at all… completely missing out on not only what … [palliative care] is, but also how it could benefit them…’ (ICB-P&2) ‘…spend time in the communities themselves, which is needs to be at local, you know very local level and go out to those communities, find their leaders and say what does your community want or need in terms of palliative, end of life care’ (ICB&P1) ‘...mean it might well be the case that the palliative care that is offered is not appropriate to everybody’ (ICB&P3) ‘It’s about how we present …to various … groups, but the message ought to be more or less the same in in essence, it’s about adapting that same message to be acceptable to the particular ethnic groups and subgroups’ (ICB&P1) ‘…there’s too much information out because everybody is giving them information…provide information when it is needed. Don’t hand out information’ (PPIE-F 6)	Infographic features people of different ages and backgrounds; non-NHS colours to reflect community focus. Film shows clinical teams, physiotherapists, carers/friends working together; subtitles included.
Broader Applicability	Ensure resources/outputs are relevant across multiple settings and adaptable for different care settings (e.g. community, care homes, hospitals). Co-design guidance on applying knowledge to individual cases rather than broad labels or diagnoses.	Co-design tools that help professionals use symptom- and concern-based criteria (e.g. from PCOMs) to support timely and appropriate care, tailored to individual patients rather than condition labels.	‘…they’ll see COVID in capital letters … and they’ll dismiss it [the findings]’ (PPIE-M1) ‘it will be interesting to see if this is in any way useful to say to people in the Community, particularly places like care homes’ (ICB&P1) ‘…how universal these factors criteria are… what we’re saying here is not specific to patients with COVID and… it’s possibly … a combination of three triggers [symptoms/concerns captured by PCOM] for patients with other end of life conditions.’ (ICB&P2) ‘…we might not see COVID again or we might… but we will see people dying of breathlessness’ (ICB&P1) ‘…can we extrapolate this [evidence] into current day-to-day management of palliative patients’ (ICB&P1) ‘Take that knowledge [concept] and apply it to the person in the bed in front of them’ (PPIE-F1) ‘… criteria for whether they think this is patients at risk of dying imminently, or whether there’s they’re in a particular category that requires more urgent attention …’ (ICB&P1) ‘…we need to sort of triangulate’ (ICB&P2)	Infographic designed to be print-friendly for care homes, GP surgeries, hospitals. Film banner: “Relevant in your community, not just hospital.”
Building Capacity with Patient-centred outcomes	Co-design simple, practical training resources and tools that help staff feel confident using PCOM data to guide care. Integrate these into existing staff development programmes (e.g. GP training hubs, hospital induction, community health education)	Develop straightforward, co-designed guidance (e.g. decision support tools, formulas) that helps staff use PCOM data to identify when palliative care referral or support may be needed. Link materials (e.g. infographic, film) to training resources, and integrate into GP training hubs and frontline staff education to promote confident use of patient-centred data.	‘…more important for staff to recognize that (what findings mean) initially, because once staff recognized that they will then be able to do their work in transferring that information to the public and at the moment staff do not recognize that.’ (PPIE-F5) ‘…within each GP, GP’s have training hubs and that this is where this information needs to go as well’ (PPIE-F5) ‘…a formula that I can give very simply to frontline staff to say this will tell you whether you need to start really putting this one person on the palliative care pathway’ (ICB&P 1)	Infographic includes QR code to the project website, and both the infographic on open channels, as well as learning hubs for academics, researchers and clinicians.

## Discussion

Our study provides insights into how PPIE and stakeholder engagement can strengthen the communication of patient-centred outcomes in palliative care. The evidence we aimed to communicate was based on patient-centred outcome data collected from people severely ill and dying of COVID-19 who were seen by palliative care services during the pandemic [7, 9]. Through co-design, we developed practical outputs—an infographic and a short film—that help reframe palliative care as expert support for managing complex symptoms, not solely end-of-life care [26]. While the source evidence was COVID-specific, co-design participants emphasised the need to present findings in ways that also resonate with wider audiences and broader palliative contexts, especially given how common misperceptions delay access to palliative care across serious illnesses. Thus, the outputs were developed to reflect both the specific context of the data and the broader goal of improving public understanding of palliative care’s relevance beyond end-of-life and beyond COVID-19. Participants highlighted the importance of accessible language, inclusive formats, and actionable messages to guide timely care and improve public understanding of palliative care’s role in serious illness. This is particularly important given the limited public awareness of palliative care in the United Kingdom [27]. Our approach also has wider relevance for patient-centred outcome research, illustrating how co-design can translate complex outcome data into practical resources across different health contexts.

Our findings align with prior work showing that translating patient-centred outcome data into accessible formats promotes understanding, supports decision-making, and improves care [28]. Similar strategies—such as plain language tools in trials—have helped address challenges like under-reporting of harm and made complex evidence meaningful for patients [29, 30]. These approaches are especially critical in holistic, complex fields like palliative care and mental health, where demonstrating benefits of care can be challenging [31].

Our work reinforces the established value of PCOMs—including PROs—in improving care through early symptom identification, enhanced symptom control, and more patient-centred consultations [29, 32–35]. Crucially, it adds new insights into how these data can be communicated. By co-designing accessible, plain-language resources with patients, carers, and stakeholders, we demonstrated practical ways to translate outcome evidence into messages that promote timely care, public understanding, and shared decision-making.

PPIE and stakeholder engagement shaped not only how the evidence was interpreted but also the most effective formats for dissemination. This was critical for ensuring that messages were accessible, relevant, and meaningful to underserved communities, the wider public, and those commissioning care [26]. The co-designed outputs illustrate how complex outcome data can be turned into user-friendly tools that support access, inclusivity, and clearer understanding of palliative care’s contribution—offering a model for other fields of patient-centred outcome research.

While our study offers valuable insights, certain limitations should be acknowledged. The small sample size—particularly among senior leads—and the use of convenience sampling may limit generalisability. The online format and potential for researcher bias in data interpretation are also considerations. However, the strength of this work lies in its collaborative, layered PPIE model, which integrated experienced and new voices to ensure the research remained patient-centred and supported capacity-building. This model has potential for wider application in healthcare research. Given that the outcome evidence came from 572 patients across 25 services (most newly referred to palliative care with COVID-19) [9], further work should test whether these communication strategies and the resulting framework generalise to other palliative care populations and settings.

Our discussions with PPIE members and senior health and social care leaders helped translate complex outcome evidence into accessible messages that resonate with diverse audiences. The findings highlight how engagement can uncover the potential of PRO data to build public awareness, inform decision-making, and foster capacity for more patient-centred care. Exploring links between patient-centred and clinical outcomes could further enhance the relevance of these findings across care settings—including care homes—and extend their applicability beyond COVID-19 to other serious illnesses.

## Conclusions

This co-design study highlights the vital role of patient, public, and stakeholder engagement in effectively communicating patient-centred outcomes in palliative care. By collaborating with diverse stakeholders, we co-designed a framework that addresses misconceptions, promotes timely access, and improves care for people with serious illness. This model of co-design has potential to inform patient-centred outcome research and communication strategies in other healthcare settings and disease areas.

## Data Availability

Requests for deidentified transcripts can be made through the corresponding author. This request will be reviewed by the principal investigator (IJH) and the core PPI members (DS, RK) and granted upon reasonable request.
